# The Butantan Institute: History and Future Perspectives

**DOI:** 10.1371/journal.pntd.0002862

**Published:** 2014-07-03

**Authors:** Marcelo De Franco, Jorge Kalil

**Affiliations:** Instituto Butantan, São Paulo, São Paulo, Brazil; Yale School of Public Health, United States of America

The Butantan Institute, as one of Brazil's most prestigious scientific institutions, generates new knowledge through scientific research, develops and produces immunobiological and biopharmacological products of interest to public health, educates and trains human resources in the areas of science and technology, and seeks to stimulate scientific knowledge and understanding among the general population. With 113 years of existence marked by numerous technological advances directed towards public health issues, the Butantan Institute is considered one of the major scientific centers in the world.

**Figure 1:**
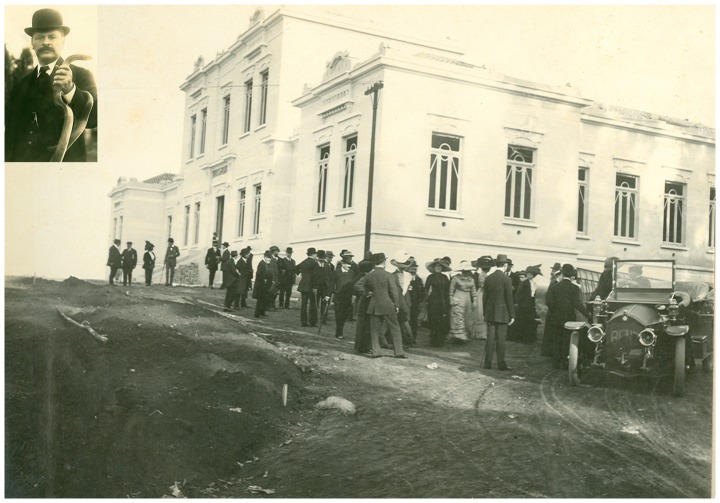
The Butantan Institute was founded in 1901, with Vital Brazil as its first director.

In 1900, a commission formed by three respected physicians and specialists in public health diseases: Dr. Emílio Ribas, director of health services for São Paulo State; Dr. Adolpho Lutz, director of the Bacteriological Institute; and Dr. Vital Brazil, an assistant of Dr. Lutz at the same Bacteriological Institute, proposed the creation of a Serum Therapy Institute in São Paulo to be installed at Fazenda Butantan, a locality distant from the state capital at that time. The foundation and direction of the Serum Therapy Institute of São Paulo State (the current Butantan Institute) was assigned to Dr. Vital Brazil Mineiro da Campanha, with the immediate responsibility of producing a serum to be used in combating the epidemic of bubonic plague afflicting the country at that time [1]. The Institute was officially established on February 23, 1901, when Dr. Vital Brazil was designated as its first director (Figure 1). The first vials of anti-bubonic-plague serum were produced in June of that same year, and the Institute has continued its work over the years in many different areas, becoming known as an important producer of several anti-ophidic serums and a groundbreaking scientific institute [2]. In parallel, Vital Brazil worked with snakebite accidents and studied venom and antibody antivenom interactions.

The evolution of the Butantan Institute can be summarized in four main periods. The administration of Vital Brazil (1901 to 1927) was accompanied by large investments in staffing, scientific research, and the construction of laboratories and an antiserum factory—in the same international context as Louis Pasteur's microbiology studies and discussions about the specificity of antivenom antibodies. Based on the correlation between the severity of the bite-site inflammation and the snake genus, Vital Brazil was the first to show that snake venom ordinarily displayed antigenic specificity. After reading a report of Calmette's anti–*Naja tripudians* serum [3], he was able to produce monovalent serums against the venoms of *Bothrops jararaca* and *Crotalus durissus terrificus*. Vital Brazil also tested and demonstrated the inefficiency of Calmette's anti-*Naja* antiserum for neutralizing *Crotalus* or *Bothrops* toxins [4]. The three pillars of success of the Butantan Institute have been preserved and strengthened since its creation: research, production, and the popularization of science.

The second phase of the Institute (1930–1970) was greatly influenced by a series of authoritarian governments, the Second World War, the organization of its pharmacology and pathophysiology laboratories, and the arrival of many foreign researchers (including Henry Slotta from the University of Breslau, Germany, who discovered the female hormone progesterone and succeeded in isolating crotoxin, the toxic protein in rattlesnake venom) [5]. The first universities and agencies promoting research in Brazil (National Council for Scientific and Technological Development [CNPq] and Foundation for Research Support of the State of São Paulo [FAPESP]) were created in the 1930s. A period of crisis later befell the Butantan Institute between 1940 and 1960, with a lack of funding and successive ineffective administrations. The National Immunization Program, created by the federal government in 1973, enabled public producers of serums and vaccines to organize and modernize their laboratories and factories, and during the 1980s, large investments were made in the Butantan Institute and the Oswaldo Cruz Foundation (FIOCRUZ) to achieve self-sufficiency in the production of vaccines and antibodies against venoms and toxins. During this period, the Butantan Foundation was created to facilitate the management of public resources, and the Biotechnology Center was established to develop new vaccines and serums. Two very successful developments can be cited for this period: the development of the hepatitis B vaccine and the modernization of the production processes for sera against poisons and toxins. This period also saw the initiation of partnerships with the private sector, including technology transfers for the production of influenza vaccine at Sanofi Pasteur. Regulatory frameworks for public health were initiated in Brazil starting in the year 2000, but public laboratories did not adjust to these legislative mandates, and a scarcity of resources (due to national and international economic crises) handicapped the leading research and production institutes, including the Butantan Institute. A major fire in the zoological collections building in 2010 was a huge loss to the scientific community.

In 2011, we assumed direction of the Institute; we then promoted a series of governance studies and developed a master plan for the expansion and professionalization of the administration of the Institute and its Foundation in order to better coordinate both. Our main goals were to establish a new organizational chart for the Institute, enhance public-private partnerships, and intensify international exchanges.

**Figure 2:**
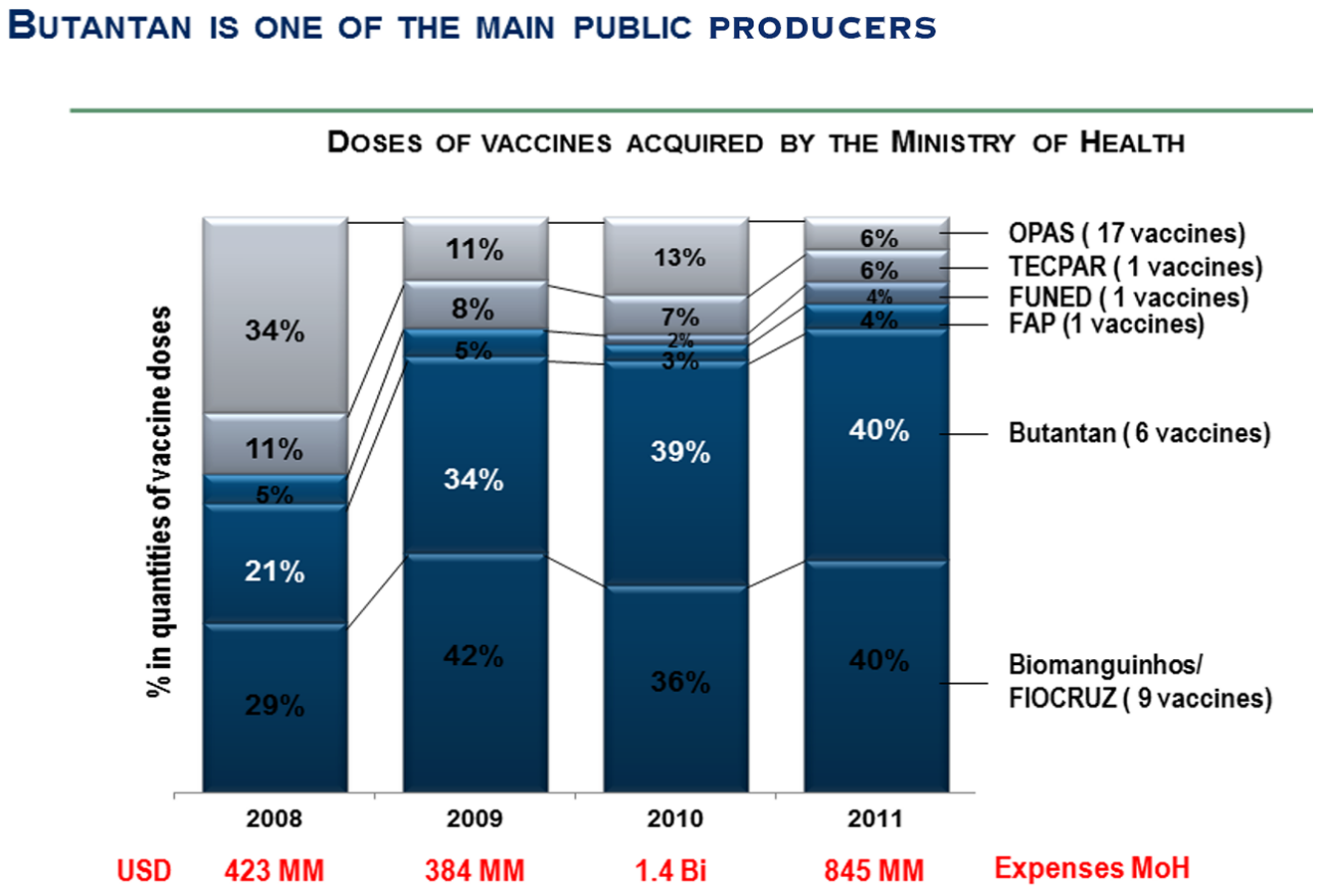
The Butantan Institute is one of the main public producers of serums and vaccines in Brazil. Abbreviations: Bi, billion; FAP, Fundação Ataulfo de Paiva; FUNED, Ezequiel Dias Foundation; MM, millions; MOH, Ministry of Health; OPAS, Organização Panamericana de Saúde; TECPAR, Institute of Technology of Paraná.

Today, the Butantan Institute, linked to the secretary of health of São Paulo State, has the mission of developing biological products for public health, undertaking basic and applied research, and promoting scientific knowledge. The Institute currently provides 40% of the nationally produced serums and vaccines that are distributed without cost to the entire population of the country by the Brazilian Health Ministry (Figure 2) through the Unified Health System (SUS).

World renowned for its studies of poisonous animals and the venoms and toxins they produce, the Butantan Institute has always attracted scientific leaders who, together with researchers and postgraduate students, develop research projects in many different areas. Isaias Raw, Antonio Camargo, Willy Beçak, Ivan Motta, Wilmar Dias da Silva, Luiz Trabulsi, and Maria Siqueria, among others, have developed projects related to vaccine production [6]–[9]; the biology and systematic classification of serpents, arthropods, and parasites; the biochemistry and pharmacology of venoms and their components; the physiopathology of venoms [10]–[15]; immunology in response to exposure to venoms and pathogenic microorganisms; the genetic basis of immune responses; and the cytogenetics and genetics of poisonous animals [16], and a number of important reviews have been published in those fields [17]–[24].

**Figure 3:**
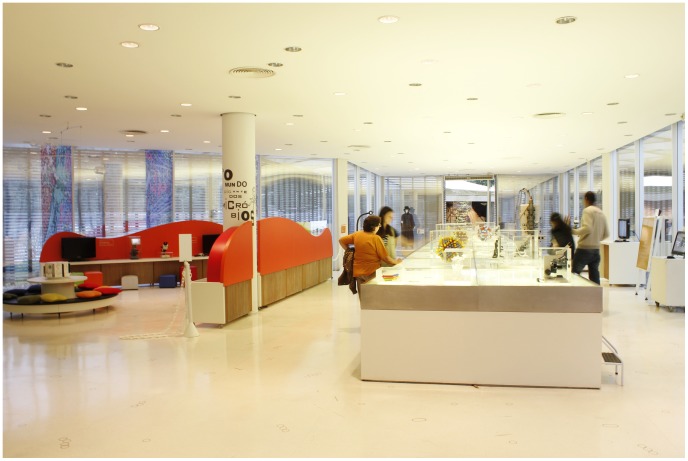
The Butantan Institute Cultural Center—Microbiology Museum.

The Butantan Cultural Development Center counts among its activities intellectual diffusion and research based on education, museology, and the history of science and public health and focuses on projects promoting scientific discoveries generated within the Institute, providing material for consultation in its documentation nucleus and library, and creating museums and educational programs. The Cultural Development Center has four museums—Emilio Ribas, Biological, Microbiological (Figure 3), and Historical—and is likewise responsible for coordinating temporary and itinerant exhibitions that attract more than 300,000 visitors each year.

**Figure 4:**
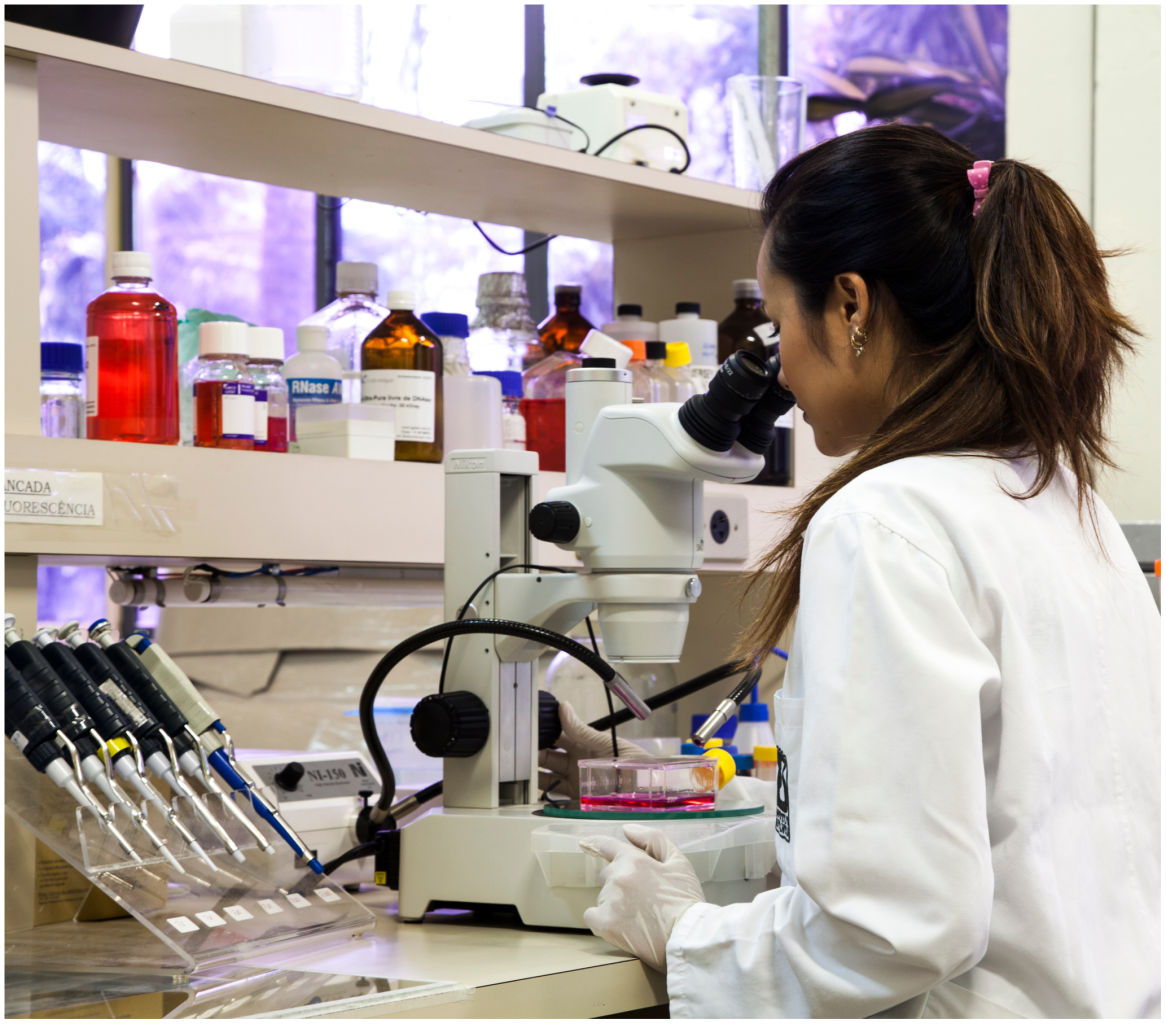
Scientific research and development laboratory at the Butantan Institute.

The Research and Production Centers of the Butantan Institute include 35 scientific laboratories, a Center for Technological Innovation, a specialized hospital (Hospital Vital Brazil), three animal facilities (one each for mammals, spiders, and serpents), seven vaccine production centers (including one for veterinary use), one center for plasma fractioning, and 11 bioproduct manufacturing sites. These research and production centers employ approximately 191 researchers, with 420 additional master's, doctoral, and postdoctoral students who undertake scientific missions within the country and throughout the world through the auspices of the World Health Organization (WHO), the Pan American Health Organization (PAHO), the United Nations Children's Fund (UNICEF), and the United Nations (UN) (Figure 4). Fully focused on the development of scientific research and the production of immunobiologicals used in public health campaigns, the Butantan Institute produces publications available for unrestricted consultation in all of its areas of action and offers internships as well as extension and postgraduate courses (MS, PhD, and MBA).The Butantan Institute has two PhD courses: Toxinology and Biotechnology (the latter offered in association with the University of São Paulo).

**Figure 5:**
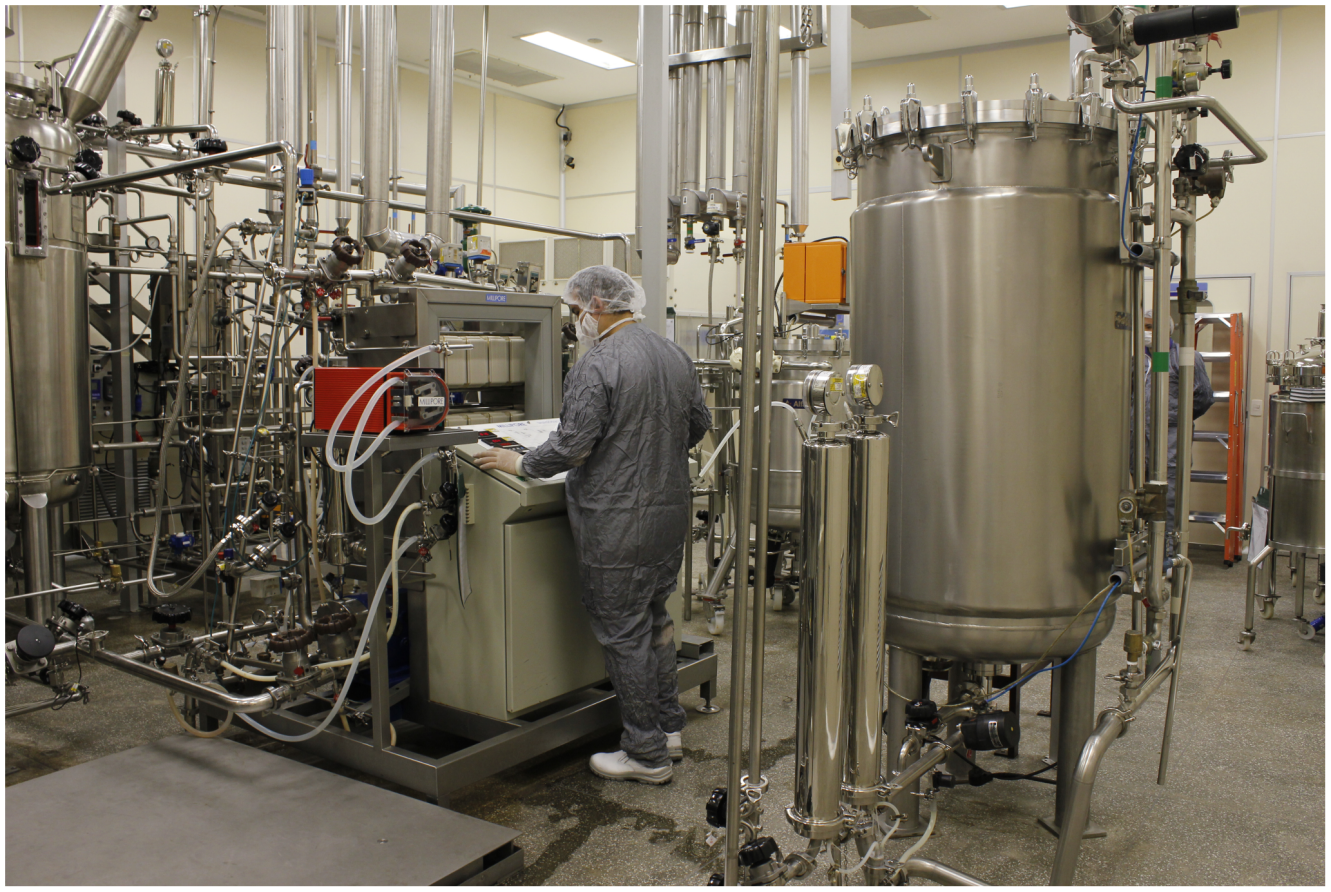
The Butantan Institute industrial complex.

The productive complex of the Institute has dominated the technologies required for producing at least 12 types of serums and seven vaccines (Figure 5) utilized by the Brazilian Health Ministry [25] and has been working with technology transfer from public and private producers in industrialized countries as well as on its own independent production innovation and the development of technologies for vaccine production.

Technology transfer is a complex, multifaceted, and delicate process that involves not only transferring basic knowledge but also techniques for quality control and quality guarantees, the domination of regulatory processes, clinical studies to guarantee the adequacy of the products, and the capacity to adapt to local conditions, all done while renovating and updating production facilities to international quality standards. The Butantan Institute industrial complex was installed in 1998 and inaugurated in 2007; it is capable of producing vaccines against various influenza virus subtypes, such as H1N1, swine flu, H5N1, and avian flu. Just four years later in 2011, the Institute delivered the first lot of vaccines against influenza entirely produced in Brazil and received a certificate of good production practices from the Brazilian National Agency of Public Health (ANVISA) in 2012. This was the first successful technological transfer completed in Brazil between Sanofi-Pasteur and the Butantan Institute [26]. Three new agreements were recently signed between international laboratories and the Butantan Institute for the development and production of vaccines against human papillomavirus (HPV) and hepatitis A with Merck Sharp & Dohme (MSD) and against acellular pertussis with GlaxoSmithKline (GSK) through technology transfer.

Additionally, the Butantan Institute independently produced a trivalent vaccine against diphtheria, tetanus, and pertussis (whooping cough) as well as a vaccine against hepatitis B in the 1980s [25].

A number of international agencies have demonstrated interest in Brazil in terms of producing and furnishing vaccines, with demand from Colombia and some African countries for technology transfers and collaboration agreements to produce Butantan antiserums. Additionally, researchers from the Butantan Institute are working through grants provided by financing agencies (FAPESP, Coordination for the Improvement of Higher Education Personnel (CAPES), CNPq, the Brazilian Development Bank (BNDES), and the Fulbright Foundation) on various projects in cooperation with scientists from various international institutions [27].

The technical competency of the Butantan Institute and its investments in upgrading its production facilities to both national and international standards (the Food and Drug Administration [FDA], WHO, and ANVISA) has stimulated interest in partnerships with international universities and other institutes dedicated to public health. Examples of these partnerships and developing projects include collaboration with the National Institute of Health (NIH), for the production of vaccines against rotavirus and the dengue virus; the Boston Children's Hospital at the Harvard Medical School, for developing a vaccine against pneumococcus; the Sabin Institute and George Washington University, for developing a vaccine against schistosomiasis (the parasites *Necator* and *Schistosoma*); and the Infectious Diseases Research Institute in Seattle and the University of Washington, for developing a vaccine against canine leishmaniasis.

The prestige of the Butantan Institute in the area of toxins produced by animals and microorganisms was decisive in its hosting of one of the Centers for Research, Innovation and Diffusion supported by FAPESP since 2002—the Center of Applied Toxinology. This program was developed to fund institutions with proven capacity in attaining world-class research levels. The initial program was quite successful, and FAPESP approved a new challenge in 2013—the Center for Research in Toxins, Immune Responses, and Cellular Signaling—that would concentrate on studies concerning the biochemical, molecular, and cellular action mechanisms of toxins demonstrating therapeutic potential, with the objective of establishing proof of concept based on the analyses of molecular signaling networks. Strategic planning calls for the results of these research projects to be transferred to industry through processes mediated by the Technology Innovation Office of the Butantan Institute.

The Butantan Institute is currently proposing the creation of the Butantan Institute for Biotechnological Innovation (IIBB) to use its accumulated technical-scientific experience in an institutional management system designed to promote agility in the administration of innovation. An international committee composed of renowned scientists is currently being formed that will analyze all of the research lines of the Institute and, together with the directory, propose a strategic plan for the next ten years and reorganize all of the research groups in the IIBB that are involved in research and development. This is one of the Institute's most important programs for the future, as it is designed to promote interactions with private partners for the development of its discoveries and innovations and the incorporation of new products into actions directed toward public health. This program proposes a new juridical model (special autarchy) for the Institute, designed to achieve autonomous administrative governance, combining Institute and Foundation to promote scientific research, technological development, and the production of vaccines and immunobiologicals in Brazil.

Our greatest efforts will always be directed towards maintaining the highest levels of excellence in research, development, and the production of vaccines and serums and guaranteeing the position of the Butantan Institute as a bridge between research and production so that Brazil will stay at the forefront of progress in public health considerations.
